# Alpha-Synuclein Induced Immune Cells Activation and Associated Therapy in Parkinson’s Disease

**DOI:** 10.3389/fnagi.2021.769506

**Published:** 2021-11-05

**Authors:** Ruichen Su, Tian Zhou

**Affiliations:** ^1^Queen Mary School of Nanchang University, Nanchang University, Nanchang, China; ^2^School of Basic Medical Science, Nanchang University, Nanchang, China

**Keywords:** Parkinson’s disease (PD), α-synuclein, microglia, T cells, therapeutics

## Abstract

Parkinson’s disease (PD) is a neurodegenerative disorder closely related to immunity. An important aspect of the pathogenesis of PD is the interaction between α-synuclein and a series of immune cells. Studies have shown that accumulation of α-synuclein can induce an autoimmune response that accelerates the progression of PD. This study discusses the mechanisms underlying the interaction between α-synuclein and the immune system. During the development of PD, abnormally accumulated α-synuclein becomes an autoimmune antigen that binds to Toll-like receptors (TLRs) that activate microglia, which differentiate into the microglia type 1 (M1) subtype. The microglia activate intracellular inflammatory pathways, induce the release of proinflammatory cytokines, and promote the differentiation of cluster of differentiation 4 + (CD4 +) T cells into proinflammatory T helper type 1 (Th1) and T helper type 17 (Th17) subtypes. Given the important role of α-synuclein in the immune system of the patients with PD, identifying potential targets of immunotherapy related to α-synuclein is critical for slowing disease progression. An enhanced understanding of immune-associated mechanisms in PD can guide the development of associated therapeutic strategies in the future.

## Introduction

Parkinson’s disease (PD) affects more than 3% of the population aged 65 years and older and is the second most common neurodegenerative disease globally (Ascherio and Schwarzschild, 2016; Bonam and Muller, 2020). PD is an enormous economic burden for the patients and society. The cost incurred by a single patient with PD in the United States (US) is estimated more than $10,000 (Kowal et al., 2013; Yang W. et al., 2020). The main manifestations of PD are motor symptoms such as tremor, rigidity, bradykinesia, and dyskinesia and non-motor symptoms such as autonomic dysfunction, cognitive abnormalities, and emotional problems (Jankovic, 2008; Palmeri et al., 2017). Epidemiology studies have shown that genetics along with environmental factors such as gut microbiota, pesticides, and heavy metal contribute to the pathogenesis of PD (Scheperjans et al., 2015; Pietrucci et al., 2019). The major pathological event in PD is the death of dopaminergic neurons in the substantia nigra pars compacta and the accumulation of α-synuclein (Surmeier, 2018), which is predominantly observed at the presynaptic terminals. Previous study has reported that α-synuclein can be released from the neurons in human model system (El-Agnaf et al., 2003; Henderson et al., 2019). Autophagy, metabolic disorder, and neuroinflammation have been proposed as the mechanisms linking α-synuclein deposition to neuronal death. For example, defects in autophagy result in abnormal α-synuclein clearance (Park et al., 2020) and recent studies have reported that the dysregulation of interactions among endoplasmic reticulum stress, unfold protein response, and autophagy are observed in the pathogenesis of PD (Ren et al., 2021). Besides, many convinced results also reported that mutations within autophagy-related genes such as PTEN induced putative kinase 1 (*PINK1*), *PARKIN*, *PARK7* (*DJ-1*) play an essential role in mitochondrial dysfunction and the dysfunction of mitochondria can successively induce a series of metabolic disorders (Kasten et al., 2018). However, the production of autophagy and mitochondrial dysfunction such as inducible nitric oxide (iNO), reactive oxygen species (ROS), and abnormal accumulation proteins can all involve the activation of central nervous system (CNS) immune system and chronic neuroinflammation that result in PD (Marogianni et al., 2020).

In CNS, microglial cells mainly act as macrophages in the brain parenchyma; other minor peripheral infiltration immune cells such as T cells, dendritic cells, and B cells are located in protective covers of the brain and most of them are generally in the resting state (Engelhardt, 2008). However, abnormal immune system activation is found in PD (Baird et al., 2019; Thomas Broome et al., 2020). Typically, researchers found that the inflammatory process in PD is mainly induced by microglia that helps in the release of cytokines. Besides, it was also reported that in the pathogenesis of PD, both the innate and adaptive immune responses are essential for the inflammation of neuronal cells (Kannarkat et al., 2013; Chen et al., 2018; Schonhoff et al., 2020). For instance, cluster of differentiation 8 + (CD8 +) and cluster of differentiation 4 + (CD4 +) T cells are detected in both the postmortem brain tissue from the patients with PD and mouse models of PD (Brochard et al., 2009).

Simultaneously, α-synuclein accumulation and the formation of Lewy bodies (LBs) in dopaminergic neurons contribute to the inflammatory mechanism in PD (Baba et al., 1998; Wang et al., 2020). PD-associated α-synuclein accumulation is reported to increase in the peripheral plasma and cerebrospinal fluid (CSF). Abnormally increased α-synuclein might be closely related to the abnormal activation of the central and peripheral immune system that is also revealed to affect the pathological mechanisms of the microglia and T cells (Olesen et al., 2018). Above all, clarifying the relationships among these factors is critical for understanding the role of neuroinflammation in the development and pathogenesis of PD.

**FIGURE 1:**
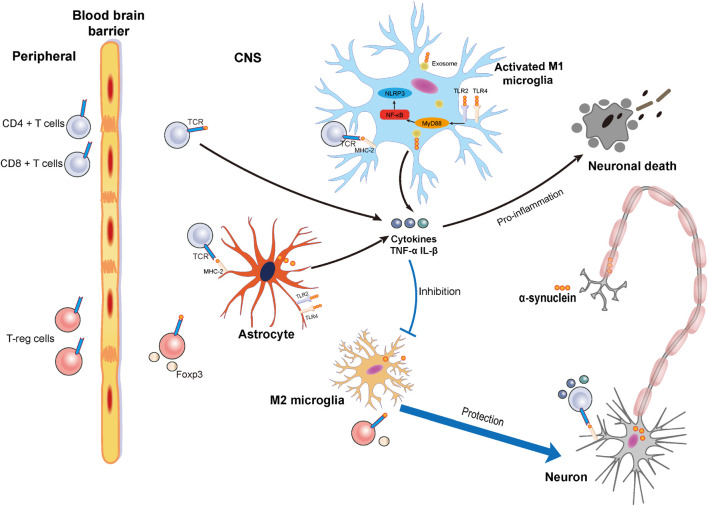
Schematic illustration of neuronal death and protection induced by the activation of microglia via α-synuclein and the relationship between α-synuclein and T cells. Accumulated α-synuclein released from neurons binds the Toll-like receptor 2 (TLR2) or Toll-like receptor 4 (TLR4) to induce a proinflammation cascade. At the same time, nuclear factor-kappa B (NF-κB) is activated to induce the MyD88 pathway for M1 microglia activation. α-synuclein can also be transferred into microglia by exosome. Proinflammatory cytokines such as tumor necrosis factor-α (TNF-α) and interleukin-1β (IL-1β) can be released from activated M1 microglia to result in neuronal death. Damage to the blood–brain barrier (BBB) in Parkinson’s disease (PD) results in the infiltration of T cells including cluster of differentiation 4 + (CD4 +) T cells, cluster of differentiation 4 + (CD8 +) T cells, and regulatory T cells from the peripheral blood that can infiltrate into central nervous system (CNS). In the CNS, microglia and astrocyte act as the antigen-presenting cells (APCs) to present the α-synuclein to the T cells. Activated CD4 + and CD8 + T cells release cytokines that promote neuronal death, while Tregs exert a protective effect on neurons.

In this study, we discuss the contribution of neuroinflammation to PD including the effect of α-synuclein on the microglia and T cells (Figure 1). We will also discuss some potential immune-based therapeutic strategies that target α-synuclein.

## Effect of α-Synuclein on Microglia

### Overview of Microglia

Since the first report of activated microglia in postmortem brain tissue samples from the patients with PD, microglia have been implicated in the neurodegenerative diseases such as PD and Alzheimer’s disease (AD) (McGeer et al., 1988; Streit et al., 2004; Gerhard et al., 2006). Microglia can act as the macrophage in the CNS to protect against pathogens and regulate the homeostasis in the brain (Casano and Peri, 2015; De Schepper et al., 2020). The subtype of microglia consists of M1, which mainly functions in the first line to clear the pathogens and M2, which is generally considered to inhibit the proinflammatory responses with an increase to repair in gene expression (Tang and Le, 2016). In the resting state, a small number of microglia can be activated to clear the pathogens or abnormal proteins and, thereby, maintain homeostasis. However, abnormal protein deposits and increase in ROS can induce the activation of M1 microglia. During the progression of PD, abnormal α-synuclein aggregated is phagocytosed, stimulating the inflammation response. A previous study demonstrated that microglia-mediated neuroinflammation affects dopamine neurons (DNs) survival in the patients with PD, implying that it plays an important role in the pathogenesis of PD (Ouchi et al., 2005). A recent study also revealed that microglia exert this effect via the regulation of DJ-1 (Lin et al., 2021). Besides, a recent comment also introduced the mechanisms of α-synuclein handling by microglia (Song et al., 2021). Consequently, the interaction between the microglia and α-synuclein is a critical aspect of the pathogenesis of PD.

### Inflammatory Pathway of Microglia Induced by α-Synuclein

Toll-like receptors (TLRs) and the NOD-, LRR- and pyrin domain-containing protein3 (NLRP3) inflammasome are involved in the activation of microglia. Other factors such as senescence and gut microbiota can also influence the function of microglia.

One essential component that facilitates the activation and interaction of microglia with α-synuclein is the TLR. As reported, TLR4 in microglia contributes to the generation of ROS induced by α-synuclein and proinflammatory cytokines (Qin et al., 2005; Fellner et al., 2013). Simultaneously, as a type of pattern recognition receptor (PRR), α-synuclein can bind to TLR2 to active microglia, which was described as α-synuclein induced non-cell autonomous neurotoxic effects of microglia (Kim et al., 2013, 2016). There are also other studies indicate that the proinflammatory cytokines could be directly activated by higher-order oligomeric α-synuclein by binding to TLR1/2 and it was achieved by nuclear factor-kappa B (NF-κB) translocation and tumor necrosis factor-α (TNF-α) induction via a Myeloid differentiation primary response 88 (MYD88)-dependent pathway (Daniele et al., 2015).

Activation of NLRP3 inflammasome is also considered an important pathogenic mechanism in microglia (Swanson et al., 2019). It was revealed that α-synuclein could act as a signal to promote the NLRP3 inflammasome assembly in microglia (Lu and Hu, 2012). Furthermore, activated NLRP3 inflammasome will also increase the accumulation of α-synuclein. Another study also demonstrated that the downregulation of the NLRP3 inflammasome components reduces the expression of the cytokines interleukin (IL)-1β and IL-18, implying that it contributes to cytotoxic microglia regulation (Javed et al., 2020).

### How Can α-Synuclein Activate Microglia?

Gene mutation is an essential factor affecting the activation of microglia. On the molecular level, a series of complex signal pathways and gene expressions are involved in the interaction between α-synuclein and microglia functions related to PD. For example, Fyn is a Src family tyrosine kinase that can be expressed in the brain. It is revealed as a critical signal mediator in the process of microglial-induced neuroinflammation in PD. This Fyn kinase-mediated pathway will activate NLRP3 inflammasome and consequently induce IL-1β secretion by microglia (Panicker et al., 2015, 2019; Nalls et al., 2018; Kam et al., 2020). Besides, the lack of PINK1 will increase the stimulation of α-synuclein by microglia to produce more NO to provoke immune response and death of neurons (Sun et al., 2018). To further understand the molecular mechanisms of α-synuclein associated immune-mediated neurotoxicity, Sarkar et al. analyzed the proteomics of α-synuclein-mediated microglial activation in microglia of mouse. It was confirmed that the expression of 864 genes such as *Irg1*, *Ifit1*, and *Pyhin* increased during the activation of microglia. In addition, they also observed that some critical proteins such as Cdc123, Sod1, and Grn, which play an important role in the different processes, including metabolic and lysosomal, were decreased (Sarkar et al., 2020).

Recently, researchers have observed a series of metabolic changes combined with the activation of microglia to satisfy the energy requirement of activation of inflammatory cells. A mounting of studies had revealed that metabolic reprogram provides critical power to activation of microglia. In this process, increased glycolysis and decreased fat metabolism were shown to be closely associated with the regulation of α-synuclein (Ganeshan and Chawla, 2014; Yang et al., 2021). A recent study has revealed that α-synuclein can bind pyruvate kinase M2 (PKM2) directly to promote the glycolysis and the migration of microglia. In this case, microglia will be converted to M1 proinflammatory type to increase the inflammatory reaction (Qiao et al., 2019).

The above studies demonstrate that besides the direct activation of the inflammation pathway, α-synuclein can also regulate the activation of microglia via the abnormal expression of proteins caused by gene mutation or through metabolic regulation. These lines of evidence confirm the association between α-synuclein and activation of microglia in PD.

### Relationship Between Activation of Microglia and Deposition and Transmission of α-Synuclein

#### Senescence of Microglia and Deposition of α-Synuclein

Given that PD is strongly related to aging, the senescence of microglia is an important factor in the pathogenesis of PD. A previous study has demonstrated that senescent microglia release TNF-α to promote the deposition of α-synuclein (Sierra et al., 2007). Besides, Angelova and Brown found a method to establish a senescent microglia phenotype module *in vitro* based on iron overload. They observed that the secretion of TNF-α by microglia was increased, which could affect the function of α-synuclein and increased its expression and aggregation levels (Angelova and Brown, 2018). Thus, the senescence of microglia may affect the deposition of α-synuclein in PD.

#### Alpha-Synuclein Transmission Through Internalization of α-Synuclein-Containing Exosomes by Microglia

It has been hypothesized that the prion-like spread of α-synuclein in the brain promotes the progression of PD (Stopschinski and Diamond, 2017; Steiner et al., 2018). To investigate how this process is regulated by microglia, George et al. developed mouse models to deplete or activate the cells of microglia to evaluate the effect on the transfer of hu-α-syn. This resulted in the accumulation of huα-syn in grafted dopaminergic neurons with fewer cells of microglia. These results demonstrated that active state regulation might influence the transfer of α-synuclein, which indicates the possibility of controlling the neuropathology expansion of PD (George et al., 2019). Besides, the state of microglia can be regulated by exosomes and affect α-synuclein. This pathway can be observed both in the central and peripheral nervous systems. To confirm the conclusion, Yun Xia et al. injected plasma exosomes from the patients with PD into the striatum of the brains of mice to evaluate exosome function in the transmission of α-synuclein. They demonstrated that microglia could uptake plasma exosomes effectively. The immunofluorescent costaining showed that the microglia released exosomal α-synuclein, which could promote exosomal α-synuclein to transfer to neurons (Xia et al., 2019). This result suggested that microglial cells can also play a critical role in the transmission of α-synuclein through the exosomal pathway. In addition, it was also considered that the progression of PD could be altered by the regulation of exosome secretion and microglial activation state. Therefore, senescence can activate microglia, which promote the transmission of α-synuclein via exosomes in the progression of PD.

Taken together, the evidence to date demonstrates that the interactions between microglia and α-synuclein in PD are related to the factors including recognition of PRR, multiple signal transduction pathways, metabolic regulation, senescence of microglia, and exosome secretion. These factors play an essential role in the neurotoxic effect of microglia in PD and are potential biomarkers of the progression and therapeutic targets of PD.

### Neuroprotective Effect of Microglia and α-Synuclein

Although the neurotoxic mechanism of microglia has been demonstrated, there is also evidence for their neuroprotective function. A study observed that removing microglia can increase the accumulation of α-synuclein, which suggested that the important function of microglia might clear the α-synuclein from the extracellular space and indicated the potential function of neuroprotective by microglia (George et al., 2019). One of the interesting hypotheses of the neuroprotective mechanism of microglia is removing extracellular α-synuclein and protecting neurons through selective autophagy. This process is regarded as a possible mechanism of synucleinphagy. Choi et al. identified that microglia activated by α-synuclein could engulf α-synuclein into autophagosomes to be degraded by synucleinphagy, a type of autophagy. They also demonstrated that TLR4-NF-κB-p62 mediated synucleinphagy that could clear the α-synuclein to realize the neuroprotective function of microglia (Choi et al., 2020a, b). The study focused on human genomics has revealed that the autophagy-lysosome system was critical in the pathogenesis of PD (Chang et al., 2017). Therefore, strategies targeting the regulation of α-synuclein homeostasis in CNS can promote the neuroprotective function of microglia in the patients with PD.

## Effect of α-Synuclein on T Cells

### Overview of T Cells

T cells play a critical role in the adaptive immune system by clearing pathogens such as bacteria and viruses. During infection, naïve T cells are activated, expanded, and differentiated into effector T cells that have an anti-infection function. The phenotype of differentiated T cells consists of CD8 + T cells, which is cytotoxic “killer cells” to kill the infected cells directly; CD4 + T cells, which act as “helper cells” to regulate the death of cells indirectly; and regulatory T cells (Treg), which is important in immune tolerance. Usually, T cells are not likely to infiltrate into CNS because of the immune privilege of the blood–brain barrier (BBB). However, T cells can infiltrate CNS and regulate neurons when BBB is damaged (Mundt et al., 2019). In the recent decades, evidence shows the significant relationship between activation of T cells and neurodegenerative diseases, especially AD and PD (Ethell et al., 2006; Yang Q. et al., 2020). Consequently, the mechanism of different subtypes of T cells is an important problem that can help to clarify further immunological mechanisms of PD and build targets for PD therapy. There are few reports about the mechanism of T helper type 2 (Th2) cells related to PD and the details of Th2 will not be included in this study. In the following sections, we discuss the role of T lymphocytes in the pathogenesis of PD.

#### CD8 + T Cells

As a “killer T cell,” CD8 + T cells can be divided into CD8 + effector T cells and CD8 + memory T cells that can directly bind antigens. Previous reports observe that the percentage of CD8 + T cells is predominantly increased among the peripheral blood in PD (Baba et al., 2005). This alternation reflects that CD8 + T cells may participate in the pathogenesis of PD. To identify the conclusion, Jordi et al. exploited immunohistochemistry and immunofluorescence to investigate the state of CD8 + T cells infiltration in the progression of PD. The results showed that in the earliest stage, a robust CD8 + T cells infiltration with no dopaminergic neuronal death in the absence of α-synuclein. However, it was observed that CD8 + T cells cause infiltration with an accumulation of α-synuclein and neuronal death throughout the later stages. Therefore, these evidence indicated that the accumulation of α-synuclein and neuronal death are associated with the activation of CD8 + T cells (Galiano-Landeira et al., 2020).

#### CD4 + T Cells

CD4 + T cells will differentiate into activated subtypes including proinflammatory T helper type 1 (Th1) and T helper type 17 (Th17) cells and anti-inflammatory Th2 and Treg upon interaction with the antigen-presenting cells (APCs). These activated subtypes will migrate in the brain to achieve their function. Kustrimovic et al. investigated the level of CD4 + T cell subtypes in peripheral blood of the patients with PD without therapy (Kustrimovic et al., 2018). The results show decreased circulating naïve CD4 + T cells with the increased percentage of Th1, IFN-γ, and TNF-α cells. Additionally, the percentage of Th2 and Treg cells decreases due to Th1 bias (Kustrimovic et al., 2018). Inferring the mechanisms underlying these changes is a promising strategy for treating PD.

### Effect of CD4 + T Cells on Neuron

#### Proinflammatory Cells Th1 and Th17

As reported, the proportion of Th1 cells is increased in the peripheral blood of the patients with PD and cytokines such as TNF-α secreted by the activation of Th1 might affect neurons (Pennock et al., 2013; MacMahon Copas et al., 2021). Th1 cells enhance inflammation by promoting the secretion of cytokines. However, it remains unclear how Th1 cells contribute to the pathogenesis of PD. More studies about it are necessary in the future.

The number of circulating Th17 was found to be elevated in the early stage of PD (Chen et al., 2017). Proinflammation Th17 cells are thought to mediate the pathology of PD by inducing the upregulation of cytokines such as IL-6, IL-23, and IL-1β (Manocha et al., 2017). Th17 cells can also directly induce the apoptosis of dopaminergic neurons by binding to some molecular proteins in the membrane such as leukocyte function-associated antigen (LFA)-1 and intercellular adhesion molecule (ICAM)-1 interaction that are activated to induce dopaminergic neuronal death to promote PD (Liu et al., 2017). In addition, compared to the normal cells, neurons of midbrain derived from PD-induced pluripotent stem cells (iPSCs) demonstrate the mechanism of pathogenesis via the increase of IL-17–IL-17R signaling and NF-κB expression associated with the activation of Th17 (Sommer et al., 2018). This evidence approved the Th17 proinflammatory role and its related signal pathway that make the critical effects on dopaminergic neuronal death. Therefore, inhibiting Th17 or its related function may have a neuroprotective effect in PD.

#### Protective Effect of the Treg

Regulatory T cells is CD4 + CD25 + T cell that can express Forkhead box (FOX)P3 to maintain self-tolerance and prevent autoimmunity (Sakaguchi, 2004). In PD, Treg is indicated to play an important role in suppressing the function of effector T cells to prevent neuronal death. Jessica A. et al. evaluated the association between the progression of PD and the state of Treg and demonstrated that the immune suppression and neuroprotective function of Treg cells were impaired in the patients with PD (Saunders et al., 2012). This result suggests the neuroprotection of Treg in PD. Further research focused on the mechanism of protection of Treg. Yan et al. built mice models with the preparation of Treg in ventral mesencephalic (VM) neurons before treating 1-methyl-4-phenylpyridinum (MPP +) to investigate the neuroprotective pathway of Treg. The results revealed the dopaminergic neurons could be protected from MPP + toxic through CD47-signal regulatory protein alpha (SIRPA) interaction and Ras-related C3 botulinum toxin substrate 1 (Rac1)/Akt (also known as protein kinase B) pathway controlled by Treg (Huang et al., 2017). This observation provides mechanistic insight into the role of Treg in PD and potential therapeutic targets.

In conclusion, CD4 + T cells play a central role in the pathogenesis of PD. Th1 and Th17 exploit multiple pathways to mediate neuronal death and promote the progression of PD. On the other hand, this neurotoxic effect can be abrogated by increasing the number of Tregs. Taken together, a more detailed exploration of the regulation of CD4 + T cells in PD is warranted.

### Alpha-Synuclein in the Relationship Between Autoimmune Response and T Cells

Alpha-synuclein acts as the neuron-encoded protein mainly involved in the transport of vesicles and neurotransmitters across the neurons. During the PD, the overaccumulation of α-synuclein can make α-synuclein become the autoantigens recognized by the immune cells. A previous study has demonstrated that α-synuclein in the hematopoietic cells is associated with the abnormal activation of the adaptive immune response in PD (Shameli et al., 2016) and α-synuclein can be presented by microglia, which acted as APCs to stimulate T cell response. Besides, a recent study demonstrates that astrocytes with accumulated α-synuclein were also shown as APCs to induce the activation of T cells and spread the inflammation (Rostami et al., 2020). Both α-synuclein and modified α-synuclein can affect the function of T cells. For example, nitrated (N) α-synuclein is revealed to induce the response of T cells and contribute to T cell-mediated neurotoxic process. This process of PD is considered to be achieved by new antigenic epitopes creation via the oxidative modification of protein, which can regulate nigrostriatal degeneration to promote the progression of pathology in PD (Benner et al., 2008; Reynolds et al., 2009). It was also reported that N-α-synuclein can modulate the neuroprotective effect of Tregs.

Stimulation of the peripheral blood samples from the patients with PD by using human-α-synuclein-derived peptide and homology herpes simplex virus1 (HSV1) peptides activated both the CD8 + T cells and CD4 + T cells compared to the healthy group (Caggiu et al., 2017). A specific type of TNF-α-secreting cell was also observed in the above stimulated blood samples. These results imply that α-synuclein affects the secretion of cytokines by T cells in the pathogenesis of PD and that infection with HSV can also promote the progression of PD. In addition to full-length α-synuclein that can induce the increase of CD8 + T and CD4 + T cells, modified α-synuclein was shown to increase its antigenicity and further stimulate the immune cell responses.

Bone marrow-derived dendritic cells (BMDCs) exert a neuroprotective effect in PD by inducing Treg. In this study, the continued costimulation with N-α-synuclein and granulocyte-macrophage colony-stimulating factor (GM-CSF) decreased the number of Tregs in a model of PD induced by 1-methyl-4-phenyl-1,2,3,6-tetrahydropyridine (MPTP). The mild costimulation on DC and Treg is demonstrated to achieve the protective effect on MPTP-PD models (Schutt et al., 2018). Consequently, we can suggest that the full-length or modified α-synuclein can affect different types of T cells to promote the progression of PD.

Major histocompatibility complex (MHC) is an essential component of adaptive immunity. In PD, overexpressed α-synuclein is an antigen recognized by MHC. Microglia MHC was shown to participate in the activation of both the innate and adaptive immune responses (Harms et al., 2013). With respect to the antigenic specificity of the adaptive immune response in PD, current evidence suggests that α-synuclein is an important target of the T-cell response and autoimmunity (González et al., 2015). Usually, α-synuclein is tolerated by T cells. However, α-synuclein will break this tolerance and can be presented to T cells to induce autoimmune response during PD. Sulzer et al. indicated the association between α-synuclein epitopes presented by specific MHC alleles and T-cell-related PD, demonstrating that the Y39 epitope of α-synuclein plays an important role in inducing T-cell response and secretion of cytokine. This research also suggests that PD results from the autoimmunity response mediated by α-synuclein (Sulzer et al., 2017). Thus, activation of autoimmunity by an aberrant accumulation of α-synuclein is an important mechanism underlying the pathogenesis of PD, although the detailed mechanism remains to be determined.

## Alpha-Synuclein as an Immunotherapeutic Target in the Patients

At present, L-3,4-dihydroxyphenylanine (L-DOPA) and deep brain stimulation are still the gold standard for the therapy of PD to relieve the motor and non-motor symptoms. However, long-term treatment with L-DOPA is not sufficient to control the symptoms that consider the newly available approach to alleviate the symptoms of PD such as immunotherapy, which exploits the specific antigen–antibody binding to regulate neuroinflammation. Given that the interactions between α-synuclein and immune cells underlie the pathogenesis of PD, immunotherapy targeting on α-synuclein is a promising therapeutic strategy. Potential immunotherapies target on α-synuclein for PD are discussed in the following sections.

### Passive and Active Immunization

Reducing the expression and aggregation of α-synuclein is an effective strategy for the treatment of PD. Previous evidence has shown that the active immunization can utilize lysosomal pathways to degenerate aggregated α-synuclein (Masliah et al., 2005). Meanwhile, anti-α-synuclein antibodies are revealed to clear the α-synuclein accumulation by activating autophagy and microglia through passive immunization (Masliah et al., 2011; Bae et al., 2012).

To confer an effective target for passive immunization antibodies, a mounting of preclinical experiments had been established. For example, Games et al. (2014) investigate the effect of monoclonal antibodies 1H7, 5C1, and 5D12 on α-synuclein in the mThy1-α-syn mouse model. The results demonstrate that the C-terminal (CT) of α-synuclein is the target of antibodies and the antibodies reduce CT-truncated α-synuclein aggregation in axons to relieve the symptoms (Games et al., 2014). This is also considered as a method to limit the transmission of α-synuclein via the sequester of extracellular protein. Besides, Weihofen et al. (2019) evaluate the characteristics and efficacy of human-derived α-synuclein antibody BIIB054 in mice of PD and report that BIIB054 plays an effective role in preventing the transmission of α-synuclein. To evaluate the safety and efficacy of the antibodies for humans, the phase 1 clinical trial of BIIB054 is established to prove the safety and tolerance in volunteers with favorable pharmacokinetics (Brys et al., 2019). With the mounting of clinical trials about passive immunization on α-synuclein is processed, monoclonal antibodies can be an effective approach for future treatment. However, because passive immunization does not induce immune memory, multiple treatments would be required. A long-lasting effect may be achieved by active immunotherapy.

PD01A and PD03A are two active vaccine candidates that induce the production of specific antibodies in CSF and plasma and can target aggregated α-synuclein. Compared with passive immunization, these two active vaccines are expected to generate long term and more specific therapy of PD. The preclinical trial has reported that these vaccines achieve their neuroprotective function, which is probably mediated by the activation of microglia and the antigen–antibody internalization to reduce the accumulation of α-synuclein (Mandler et al., 2014). A recent phase 1 clinical trial has also demonstrated that the administration of PD01A was well tolerated (Volc et al., 2020).

The above preclinical and clinical trials have demonstrated the safety and efficacy of both the passive and active immunization in the management of PD; their combination with humoral immunization is also being a potential therapeutic strategy. Rockenstein et al. (2018) designed a novo vaccination method that includes an APC-targeting glucan particle (GP) vaccine delivery system, encapsulated α-synuclein antigen, and rapamycin (RAP). This new vaccination combines cellular immunization and an active humoral system to induce both the Treg and anti-α-synuclein antibodies to boost the neuroprotective effect. The preclinical results have shown that the combination vaccine can achieve an enhanced efficacy compared to single cellular immunization (Rockenstein et al., 2018).

### Immune Modulator

Another strategy for mitigating abnormal α-synuclein accumulation in PD is to restore the balance between pro- and anti-inflammatory factors with an immune modulator. Sargramostim is an important human recombination GM-CSF approved by the US Food and Drug Administration (FDA) for bone marrow-related diseases that acts by promoting the recovery of Treg (Smith et al., 2006). In a phase 1 clinical trial, sargramostim was shown to effectively regulate Tregs to relieve the neuroinflammation in PD (Gendelman et al., 2017). Similarly, LBT-3627, a vasoactive intestinal peptide receptor 2 (VIPR2) peptide agonist, functions as an immune modulator to restore Treg function in a α-synuclein overexpressed model of PD (Mosley et al., 2019).

Recently, REXO-C/ANP/S hybrid nanoparticle system is demonstrated to clear the accumulation of α-synuclein and abnormal immune activation in PD (Liu et al., 2020). The full name of this engineered system is called rabies virus glycoprotein (RVG) peptide–modified exosome (EXO) curcumin/phenylboronic acid-poly [2-(dimethylamino) ethyl acrylate] nanoparticle/small interfering RNA targeting alpha-synuclein (*SNCA*). In this system, exosomes derived from immature dendritic cells act as a coat of the full system to promote the immune suppression. Liu et al. (2020) have shown that REXO-C/ANP/S can achieve immune activation clearing by inhibiting Th17 and enhancing Treg to regulate the immune system in mice with PD.

In summary, there is accumulating evidence that immunotherapies targeting α-synuclein are effective treatments for the patients with PD. However, more clinical trials are still needed in the future to confirm their long-term efficacy and safety.

## Conclusion

Immune mechanisms are important in the development and progression of PD, especially immune cell activation regulated by α-synuclein. This study presented the current state of knowledge regarding α-synuclein-induced microglia activation and their interactions and autoimmune responses induced by full-length proteins or modified peptides of α-synuclein. The existing evidence indicates that the interaction between α-synuclein and microglia or T cells is a “double-edged sword.” α-synuclein can induce the activation of microglia or effector T cells, leading to the production of neurotoxic cytokines and promote selective autophagy of microglia to protect neurons. How to achieve a balance of the “double-edged sword” to protect neurons is an important future direction in the research of PD.

Generally, α-synuclein is a normal functional protein that is mainly responsible for transport of vesicle and secretion of neurotransmitters. In PD, accumulation of α-synuclein makes it an autoantigen and causes an autoimmune response in immune cells. Like experimental autoimmune encephalomyelitis (EAE), an autoimmune disease with predominant activation of Th17 and immune-related therapy, it can significantly relieve this disease (Shin et al., 2018). These pieces of evidence show the close connection between the pathology and immunology of PD and PD might be a type of autoimmunity reaction-mediated disease.

Despite progress in the therapeutic strategies, PD remains an incurable disease. Therefore, we suggest that immunotherapy may also be effective in PD. As one of the biomarkers of PD, a mounting of studies reported that α-synuclein is related to immune therapy such as vaccines of PD, monoclonal antibodies, and immune modulators. The relation between α-synuclein and the immune system is expected to become a key for future treatment of PD. More detailed studies on the immune mechanism regulating α-synuclein can provide new directions for the development of treatments to slow or halt the progression of PD.

## Author Contributions

Both authors selected the related literature, conceptualized, designed, wrote, edited, and revised the manuscript.

## Conflict of Interest

The authors declare that the research was conducted in the absence of any commercial or financial relationships that could be construed as a potential conflict of interest.

## Publisher’s Note

All claims expressed in this article are solely those of the authors and do not necessarily represent those of their affiliated organizations, or those of the publisher, the editors and the reviewers. Any product that may be evaluated in this article, or claim that may be made by its manufacturer, is not guaranteed or endorsed by the publisher.
